# Targeting regulated cell death pathways in acute myeloid leukemia

**DOI:** 10.20517/cdr.2022.108

**Published:** 2023-03-15

**Authors:** Sylvain Garciaz, Thomas Miller, Yves Collette, Norbert Vey

**Affiliations:** ^1^Hematology Department, Integrative Structural and Chemical Biology, Aix-Marseille Université, Inserm U1068, CNRS UMR7258, Institut Paoli-Calmettes, Centre de Recherche en Cancérologie de Marseille (CRCM), Marseille 13009, France.; ^2^Integrative Structural and Chemical Biology, Aix-Marseille Université, Inserm U1068, CNRS UMR7258, Institut Paoli-Calmettes, Centre de Recherche en Cancérologie de Marseille (CRCM), Marseille 13009, France.; ^3^Hematology Department, Aix-Marseille Université, Inserm U1068, CNRS UMR7258, Institut Paoli-Calmettes, Centre de Recherche en Cancérologie de Marseille (CRCM), Marseille 13009, France.

**Keywords:** Acute myeloid leukemia, regulated cell death, apoptosis, ferroptosis, necroptosis, autophagy

## Abstract

The use of the BCL2 inhibitor venetoclax has transformed the management of patients with acute myeloid leukemia (AML) who are ineligible for intensive chemotherapy. By triggering intrinsic apoptosis, the drug is an excellent illustration of how our greater understanding of molecular cell death pathways can be translated into the clinic. Nevertheless, most venetoclax-treated patients will relapse, suggesting the need to target additional regulated cell death pathways. To highlight advances in this strategy, we review the recognized regulated cell death pathways, including apoptosis, necroptosis, ferroptosis and autophagy. Next, we detail the therapeutic opportunities to trigger regulated cell death in AML. Finally, we describe the main drug discovery challenges for regulated cell death inducers and their translation into clinical trials. A better knowledge of the molecular pathways regulating cell death represents a promising strategy to develop new drugs to cure resistant or refractory AML patients, particularly those resistant to intrinsic apoptosis.

## INTRODUCTION

Regulated cell death (RCD) is a biologically controlled process that differs from accidental cell death (ACD) by its reliance on defined molecular signaling pathways and tight regulation. Its well-defined nature implies that it can be modulated by pharmacological or genetic interventions contrary to ACD^[1-3]^. Schweichel and Merker were the first to report the presence of three distinct cell death morphologies: type I (apoptosis), type II cell (cell death associated with autophagy) and type III (necrosis)^[4]^. While apoptosis is the most well-known RCD, many other pathways and molecular characteristics have subsequently been described^[2]^. A better understanding of the mechanisms driving RCD may lead to the discovery of new anticancer drugs or the repositioning of older drugs to treat aggressive cancers.

Acute myeloid leukemia (AML) is a severe hematological malignancy driven by various molecular alterations and mainly occurring in adults > 60 years old. Treatment modalities of newly diagnosed AML depend on age, general conditions, comorbidities, and molecular risk factors based on cytogenetics and the presence of molecular alterations. These mutations are integrated together with cytogenetic abnormalities in the widely used and recently updated ELN 2022 classifications^[5,6]^. More than 60% of younger patients will be cured by an intensive cytotoxic therapy (ICT) induction based on the association of anthracyclin and cytarabine (7 + 3), followed by consolidations with high doses of cytarabine and/or allogeneic stem cell transplantation^[7]^. Half of the patients > 65 years cannot receive ICT because of age, poor general status, or comorbidities. In this context, the hypomethylating agents (HMA) decitabine and azacytidine (AZA) have been associated with complete response rates of 20-30% and 10-month survival^[8,9]^, highlighting the need for more effective treatments for older and not adequately fit patients.

BCL-2 inhibition is an excellent illustration of how the molecular understanding of RCD has led to the rapid development of a drug that has transformed the therapeutic treatment landscape for older AML patients. Venetoclax (VEN) in combination with the hypomethylating agent AZA has become part of standard frontline therapy for patients not eligible for ICT by improving the rates of response and overall survival compared with AZA monotherapy^[10,11]^. However, 10% to 50% of newly diagnosed patients with AML will not respond to VEN-AZA. In addition, half of the patients have relapsed by 18 months and no plateau is seen on overall survival curves. In the population of VEN-AZA refractory or relapsed patients, response rate and overall survival are poor (20% and 2.4 months, respectively)^[12,13]^. The combination of VEN with ICT is also associated with high response, but 3% to 15% of patients do not respond to the treatment^[14]^. Altogether, these data show that targeting RCD is a valuable strategy and has already improved the efficacy of the current AML therapeutic strategies. However, there is a real need to develop new drugs to go beyond BCL2 inhibition in AML^[13]^. In this review, we will discuss the main RCD pathways, describe their therapeutic targeting and discuss the main challenges for translating preclinical results into the clinic.

## REGULATED CELL DEATH PATHWAYS

### Intrinsic apoptotic pathway

**Figure 1:**
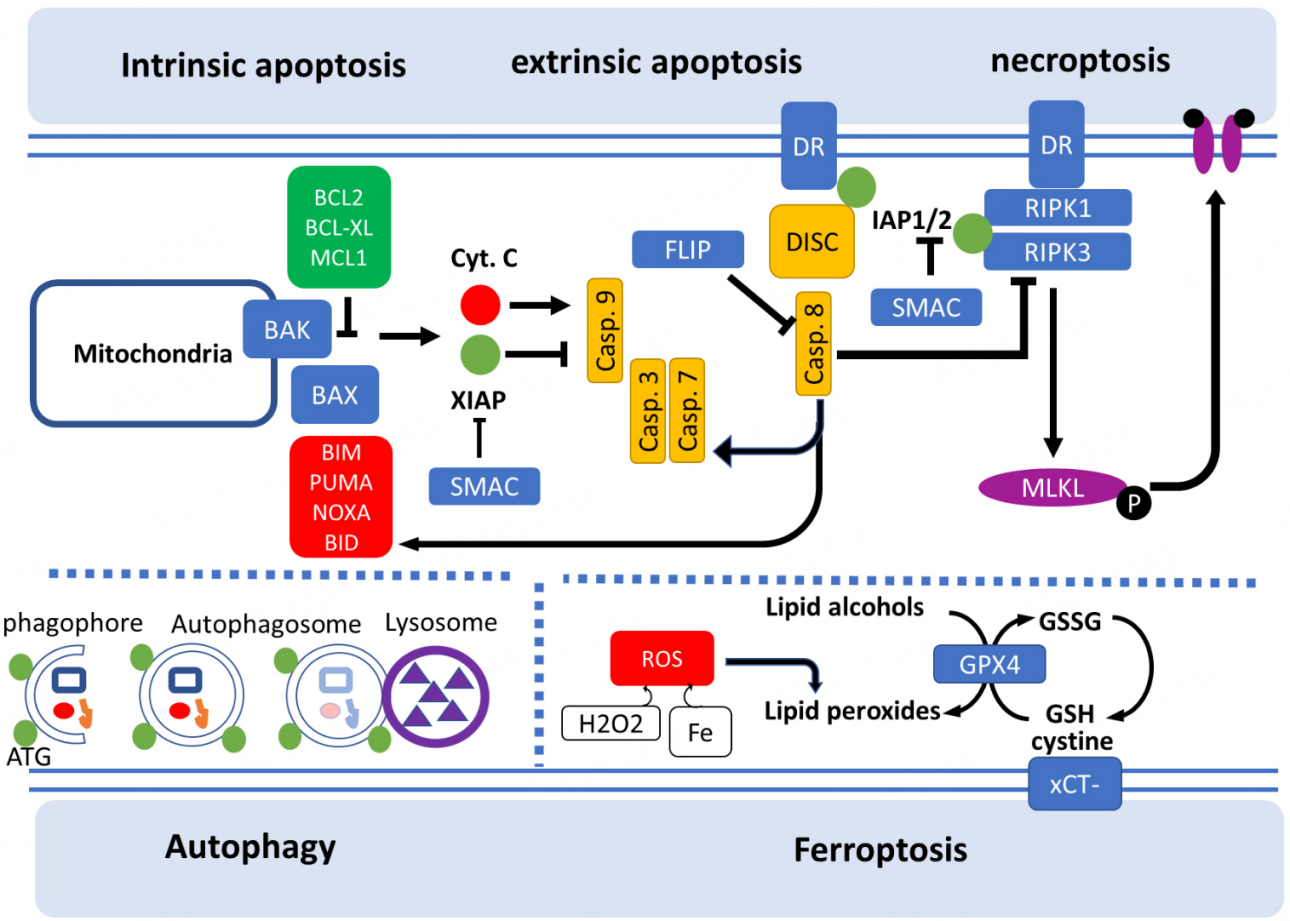
Mechanisms of regulated cell death pathways.

Proapoptotic and antiapoptotic members of the BCL2 protein family share one to four BCL2 homology (BH) domains and control intrinsic apoptosis^[15,16]^. Under physiological conditions, BCL2-associated X (BAX) resides in the cytosol in an inactive conformation while BCL2 antagonist/killer 1 (BAK) is inserted at the outer mitochondrial membrane (OMM) via an α9 helix that connects with voltage-dependent anion channel 2 (VDAC2)^[17]^. In response to apoptotic stimuli, BAX and BAK associate to form pores in the OMM, inducing mitochondrial outer membrane permeabilization (MOMP, Figure 1).

MOMP is regulated by the members of the BCL2 protein family containing a single BH3 domain named BH3-only proteins. The main representatives of this class are p53-upregulated modulator of apoptosis (PUMA), BCL2-like 11 (BCL2L11), also called BCL2-interacting mediator of cell death (BIM), phorbol-12-myristate-13-acetate-induced protein 1 (PMAIP1, also called NOXA) and BH3 interacting domain death agonist (BID)^[18]^. All these proteins have a proapoptotic role by directly interacting with BAX and/or BAK to induce MOMP. Conversely, MOMP is blocked by a series of proteins that have an antiapoptotic role. This includes BCL2, BCL2-like 1 (BCL2L1, also known as BCL-X_L_), MCL1, BCL2 like 2 (BCL2L2, also known as BCL-W), and BCL2 related protein A1 (BCL2A1, also known as BFL-1)^[19]^. Other BH3-only proteins, including BCL2-associated agonist of cell death (BAD), BCL2 modifying factor (BMF), or harakiri, BCL2 interacting protein (HRK), have the ability to induce MOMP in the absence of direct interaction with BAX or BAK by limiting the ability of the antiapoptotic BCL2 family members to sequester BAX BAK, or BH3-only activators^[20]^.

MOMP promotes the cytosolic release of cytochrome c (Cyt c) and diablo IAP-binding mitochondrial protein (DIABLO), also known as second mitochondrial activator of caspases, SMAC^[16]^. The cytosolic pool of Cyt c binds to apoptotic peptidase activating factor 1 (APAF1) and pro-caspase 9 to form the multiprotein complex called the “apoptosome” responsible for caspase 9 activation. Activated caspase 9 catalyzes the proteolytic activation of the executioner caspase 3 and caspase 7, inducing apoptotic cell death. Blocking post-mitochondrial caspase activation with Z-VAD-fmk and Q-VD-OPh caspase inhibitors delays but does not completely rescue apoptosis *in vitro* and *in vivo* as it induces a switch to other types of RCD^[2]^.

### Extrinsic apoptosis

The extrinsic apoptosis pathway is initiated by cell membrane proteins known as death receptors (DR). Proapoptotic death receptors include FAS, also known as APO1 and CD95, the tumor necrosis factor (TNF) receptors TNFR1 and TNFR2 and the TNF-related apoptosis-inducing ligand (TRAIL) receptors DR4 and DR5. DR ligation allows for the assembly of the “death-inducing signaling complex” (DISC), a multiprotein complex that activates caspase 8. At this point, the execution of extrinsic apoptosis driven by DR follows two distinct pathways. In most cancer cells, caspase 8-mediated cleavage and activation of BID converges with intrinsic apoptosis by triggering a BAX/BAK-dependent MOMP^[21]^. However, in some cells, (for instance, mature lymphocytes), the caspase 8-dependent activation of caspase 3 and caspase 7 is sufficient to promote cell death independently of mitochondria and BCL2-family of proteins^[22]^. In DR-induced apoptotic intrinsic pathway, caspases are the main executioner of cell death, causing rapid proteolysis, DNA fragmentation and chromatin condensation, which are the hallmarks of apoptosis.

Intrinsic and extrinsic apoptosis are both regulated by the class of inhibitors of apoptosis proteins (IAPs). Among the 8 IAP family members described in humans, the best known is XIAP which inhibits caspases 3, 7, and 9^[23]^. IAP proteins are antagonized by SMAC/DIABLO, which is released by mitochondria during MOMP^[24]^.

### Necroptosis

Tumor necrosis factor alpha (TNF-α) is a potent trigger for apoptosis, but it was observed that cells treated with TNF-α and the caspase inhibitor zVAD-fmk were still dying without caspase activation, suggesting the presence of an alternative RCD pathway^[25]^. In 2005, Degterev et al. coined the term necroptosis by demonstrating that this unique RCD distinct from necrosis and apoptosis^[26]^ is initiation by the receptor-interacting protein RIPK1 and could be inhibited by the pharmacological agent necrostatin-1. Indeed, necroptosis is initiated by the activation of the cell death receptors such as TNFR1 and RIPK1 (if caspase 8 is inactive) and depends on the subsequent activation of RIPK3 and the protein complex termed mixed lineage kinase domain-like pseudokinase (MLKL), resulting in the formation of pore on the cell membrane followed by cell death. Nevertheless, several of the upstream signaling elements of extrinsic apoptosis and necroptosis are shared, and sensitivity to each death pathway is regulated by the same set of regulatory molecules, including FLIP, the cellular inhibitors of apoptosis proteins cIAP1 and SMAC/DIABLO^[27]^.

### Ferroptosis

The term ferroptosis was coined in 2012 to describe a cell death that can be triggered by inactivation of the cystine/glutamate antiporter (also known as SLC7A11), leading to depletion of intracellular glutathione (GSH) or direct inhibition of glutathione peroxidase 4 (GPX4). GPX4 inhibition induces an accumulation of reactive oxygen species (ROS), ultimately leading to lipid peroxidation via the iron-dependent Fenton reaction, where H_2_O_2_ and iron react to generate hydroxyl radicals^[28,29]^. Inactivation of GPX4 through depletion of GSH with Erastin or with the direct GPX4 inhibitor RSL3 ultimately results in overwhelming lipid peroxidation that can be rescued by the use of antioxidant ferrostatin-1 or liprostatin-1 that block lipid peroxidation^[30,31]^. Ferroptosis is regulated at several levels, including amino acids, lipids (particularly polyunsaturated fatty acids (PUFA)) and iron metabolism^[32-36]^. In addition, ferroptosis is regulated by the suppressor protein 1 (FSP1) and dihydroorotate dehydrogenase (DHODH) that reduce ubiquinone (CoQ) to ubiquinol (CoQH_2_) on the plasma membrane and inner mitochondrial membrane, respectively. CoQH_2 _acts as a hyperoxide radical-trapping antioxidant and decreases lipid peroxidation, resulting in ferroptosis suppression^[37,38]^. Finally, GTP cyclohydrolase-1 (GCH1) and its metabolic derivatives tetrahydrobiopterin/dihydrobiopterin (BH_4_/BH_2_) are also potent antioxidants protecting against ferroptosis^[39,40]^. Ferroptosis is also regulated by the non-canonical activities of TP53 on cellular metabolism in a context-specific manner^[41]^. TP53 enhances ferroptosis by inhibiting SLC7A11 expression or increasing expression of spermidine/spermine N1-acetyltransferase 1 (SAT1) and glutaminase 2 (GLS2). Likewise, TP53 can inhibit ferroptosis by reducing dipeptidyl peptidase 4 (DPP4) activity or inducing cyclin-dependent kinase inhibitor 1 A (CDKN1A/p21) expression. Unlike the other RCDs, it remains to be determined whether ferroptosis is a consequence of physiological processes or necessary for development as opposed to only pathophysiological situations^[42]^.

### Autophagy

Macroautophagy (hereafter autophagy) is a ubiquitous catabolic process that involves the degradation of cytoplasmic components, including intracellular organelles, via the lysosomal pathway. The first step of autophagy is the formation of phagophores, followed by the generation of double-membrane autophagosomes regulated by a series of well-conserved autophagy-regulated genes (ATG). Late steps involved the fusion of autophagosomes to lysosomes, leading to cargo degradation by lysosomal enzymes and the recycling of intracellular contents providing fuels for cell growth^[43]^. The autophagic response provides cytoprotective effects, as indicated by the fact that blocking autophagy with pharmacological or genetic interventions generally induces cell death through the accumulation of toxic proteins, damaged organelles or undigested autophagosomes toxic for tumor cells^[43]^. Autophagy is often activated alongside other RCD, such as ferroptosis, which can be promoted by the autophagic degradation of ferritin, extrinsic apoptosis or necroptosis^[2]^. The main features of RCD are summarized in Table 1.

**Table 1:** Summary of the main regulated cell death mechanisms

**Type of RCD**	**Trigger**	**Facilitator**	**Inhibitor**	**Executioner**
Intrinsic apoptosis	Mitochondrial outer membrane permeabilization by BAX/BAK macropores and cytochrome c release	Proapoptotic factors from the BCL2 family	Antiapoptotic factors from the BCL2 family, Inhibitors of apoptosis (IAPs)	Proteolysis by executioner caspases
Extrinsic apoptosis	Activation of membrane cell death receptors	Death-inducing signaling complex (DISC). SMAC/DIABLO	Inhibitor of apoptosis (IAPs), FLIP	Proteolysis by executioner caspases
Necroptosis	Activation of membrane cell death receptors	RIPK1, RIPK3, SMAC/DIABLO	Caspase 8	Plasma membrane disruption by MLKL
Ferroptosis	Inhibition of GPX4, decrease of cystine uptake	hydroxyl radicals generation through Fenton reaction	Glutathione (GSH), antioxidant defense	Lipid peroxidation of plasma and intracellular membranes
Autophagy	Phagophore formation and fusion to the lysosome	Autophagy-related proteins (ATG)	Inhibition of lysosome acidification	Accumulation of toxic proteins or organelles leading to cellular stress

### Other cell death pathways

Pyroptosis is an inflammatory form of lytic RCD that frequently occurs in response to microbial infection by forming a multiprotein complex termed the inflammasome, which activates caspase 1 and forms Gasdermin D (GSDMD) dependent plasma-membrane pores^[44]^. Pyroptosis is mechanistically distinct from apoptosis and characterized by the absence of DNA fragmentation and the presence of nuclear condensation coupled with cell swelling. Large bubbles eventually form at the plasma membrane and rupture to expel cellular contents.

Mitotic catastrophe is a physiological mechanism by which the cells avoid aneuploidy and hence decrease tumorigenic potential. Accordingly, induction of mitotic catastrophe both precipitates oncogenesis and constitutes a therapeutic endpoint in cancer cells^[45]^.

Inter-regulation and hierarchy between cell death pathways is complex and not completely understood. For instance, mitotic catastrophe is closely related to apoptosis by their common induction by cellular stress and DNA damage. For this reason, some authors suggest that mitotic catastrophe is not a distinct mechanism of death, but one that can occur through necrosis or apoptosis depending on the molecular profile of the cells^[46]^. Other examples of cross-talk between RCD include the link between ferroptosis and autophagy, in particular through ferritin degradation or the involvement of lysosomes in iron storage and redistribution^[47,48]^. Depletion of the ferroptosis trigger GPX4 also sensitizes cells to pyroptosis^[49]^, necroptosis^[50]^ and apoptosis^[51]^. Apoptosis and necroptosis are also linked through caspase 8 cleavage [Figure 1]. Caspase 8 is also an important player in pyroptosis induction, as it has been shown that genetic lesions in XIAP result in increased inflammation and death-associated caspase-8 and GSDMD processing in diseased tissue^[52]^. Common stimuli can also result in different RCD depending on the cell type. For instance, blockage of iron transport into the lysosome can induce ferroptosis in breast cancer stem cells, whereas it induces mitochondrial BAX/BAK-dependent cell death in AML models^[53,54]^.

## RCD INDUCERS IN AML

### Apoptotic inducers

#### Agents targeting intrinsic pathway

Most chemotherapies and radiotherapies are known to induce apoptosis of cancer cells in response to DNA damage or cellular stress^[55]^. As a consequence, *in vitro *and *in vivo *evidence indicates that TP53-mutated cells have impaired apoptosis signaling pathways, and these cells are typically less susceptible to cytotoxic chemotherapy^[56]^.

Inhibitors of BCL2 family members

Besides VEN, several newly developed BCL-2 inhibitors are currently in various stages of investigation in AML and other leukemia models [Table 2]^[13,57-60]^. In addition to BCL-2, other members of the BCL-2 family of antiapoptotic proteins (MCL-1, BCL-XL, BFL-1) are the target of small molecules with the goal of inducing BAK/BAX activation and promote intrinsic apoptosis. The antiapoptotic protein MCL-1 plays a critical role in inhibiting BAX/BAK activation. MCL1 dependency on leukemia blasts is associated with resistance to BCL2 inhibition by VEN. Several highly potent direct MCL-1 inhibitors have recently entered preclinical and clinical development [Table 2]^[61-64]^. The therapeutic window of these inhibitors is narrow because of the high expression of MCL1 in cardiac and hepatic tissues^[65]^. Due to these safety concerns, indirect MCL1 inhibitors are also under evaluation. CDK9 inhibitors are in various stages of evaluation in AML [Table 2]^[66-73]^. Addition of alvocidib to ICT improved response rates but not survival in a recently published phase 2 clinical trial^[67]^. Novel CDK9 inhibitors are currently in early phase trials [Table 2]^[74-78]^. BCL-XL inhibition by navitoclax has been shown to be active in preclinical AML models^[79,80]^. Toxicity for platelets limited its clinical development; nevertheless, navitoclax in combination with ICT or targeted therapy is still under evaluation in acute lymphoblastic leukemia^[81]^ or myelofibrosis (TRANSFORM-1, NCT04472598). Navitoclax in combination with VEN and decitabine may be a valuable option for VEN-refractory AML patients (NCT NCT05222984). Finally, BFL1 (BCL2A1) inhibition may also be an interesting option since the recent discovery of specific inhibitors, but the drug has not been specifically tested in leukemia models^[82,83]^.

**Table 2:** Drugs targeting main regulated cell death mechanisms in acute myeloid leukemia

**Therapeutic class**	**Mechanism of action**	**Drug**	**Trademark**	**Phase of development**	**References**
BH3-mimetics	BCL2 inhibition by small molecule	BGB-11417	-	Phase 1 (NCT04771130)	[57]
		S65487	-	Phase 1 (NCT03755154, NCT04742101)	[58]
		APG-2575	Lisaftoclax	Phase 1 (NCT03537482)	[59]
		LP-108	-	Phase 1 (NCT04139434)	[60]
	Direct MCL1 inhibition by small molecule	S63845	-	Phase 1 (NCT02979366, NCT03672695, NCT04629443)	[61]
		AZD5991	-	Phase 1 (NCT03218683)	[62]
		AMG176	Tapotoclax	Phase 1 (NCT02675452)	[63]
		AMG397	Murizatoclax	Phase 1 (NCT03465540)	[64]
CDK9 kinase inhibitor	Indirect MCL1 inhibition by small molecule	Flavopiridol, HMR-1275	Alvocidib	Phase 1 (NCT00407966, NCT03298984), phase 2 (NCT01349972)	[66,69]
		SCH-727965	Dinaciclib	Preclinical	[70,71]
		P 1446A 05	Voruciclib	Phase 1 (NCT03547115)	[72,73]
		AZD4573	-	Phase 1 (NCT03263637)	[74]
		CYC065	Fadraciclib	Phase 1 (NCT04017546)	[75,77]
		TG02-101	-	Preclinical	[78]
BH3 mimetics	BCL-XL inhibition	ABT-263	Navitoclax	Phase 1 (NCT05222984)	-
BH3 mimetics	BFL1 inhibition	-	-	Preclinical	[82,83]
BAX/BAK activator	Direct BAX activation	BTSA1	-	Preclinical	[84]
		WEHI-9625	-	Preclinical	[87,88]
	Mitochondrial iron depletion	AM5	Ironomycin	Preclinical	[53]
TRAIL agonist	Death Receptor 5 (DR5) antibody	IGM-8444	-	Phase 1 (NCT04553692)	[89]
	TRAIL receptor agonist fusion protein	ABBV-621	Eftozanermin alfa	Phase 1 (NCT03082209)	[79,80]
FLIP inhibition	Direct FLIP inhibition	-	-	Preclinical	[92,93]
XIAP inhibition	Antisense oligonucleotide	LY2181308	Gataparsen	Phase 2 (NCT00620321)	[98]
		AEG35156		Phase 1 NCT00363974, phase 2 NCT01018069	[99,100]
	SMAC/DIABLO mimetics	TL32711	Birinapant	Phase 1 NCT01828346 phase 2 NCT01486784, NCT02147873	[102,103]
		ASTX660	Tolinapant	Phase 1 NCT02503423	[105]
		LCL161		Phase 2 (NCT02098161)	[107]
	SMAC/DIABLO mimetics + caspase 8 inhibition	TL32711	Birinapant	Preclinical	[108]
	SMAC/DIABLO mimetics	BV6		Preclinical	[109]
epigenetic therapies	Endogenous retroelements reactivation	-	Epigenetic therapies	Preclinical	[116]
Class 1 FIN	System x_c_^- ^cystine/glutamate antiporter inhibition	-	Sulfasalazine	FDA approved in another indication	[118]
Class 2 FIN	GPX4 inhibition	ML162	Altretamine	FDA-approved in another indication	[122]
Class 3 FIN	GSH metabolism inhibition	APR-246	-	Phase 2 (NCT03931291)	[124]
Class 4 FIN	CoQ oxidoreductase FSP1 inhibition	-	-	Preclinical	[37,38]
Autophagy inhibitors	Lysosomal acidification blockade	-	Hydroxychloroquine	FDA-approved in another indication	[138,139]
	PIK3C3/Vps34 inhibition	SAR405	-	Preclinical	[140,141]
Autophagy inducers	mTOR inhibition	-	Sirolimus	FDA-approved in another indication, phase 1 (NCT01869114)	[143]
		RAD001	Everolimus	FDA-approved in another indication, phase 1/2 (NCT00819546, NCT02638428, NCT01869114)	[144]

BAX/BAK activators

BAK and BAX are crucial agents in promoting MOMP through protein oligomerization across the OMM. Recent findings showed direct activation of BAX by BTSA1, a pharmacological BAX activator that binds BAX with high affinity and specificity to the N-terminal activation site and induces conformational changes to BAX, leading to BAX-mediated apoptosis^[84]^. The histone deacetylase SAHA and its derivatives also have an affinity for BAX and induce its activation [Table 2] but have not been validated in AML models^[85]^. Other preclinical studies suggested a mitochondrial membrane-mediated spontaneous model of BAX activation. In this model, MOM plays a big role in orchestrating the turnover between cytosolic and membrane-bound BAX, its interaction through the α9 helix and the formation of macropore into the membrane. It is likely that lipids such as cardiolipin play a crucial role in this model^[86]^. Voltage Dependent Anion Channels (VDACs) are a family of membrane proteins that allow passage of both negatively and positively charged ions, NADH, ATP/ADP and other metabolites across the MOM. In particular, VDAC2 plays a role both in recruiting BAK to the MOM and in inhibiting its activation^[17]^. WEHI-9625 is a novel small molecule inducing BAK-mediated apoptosis in mice but is completely inactive against human BAK^[87,88]^. We found that ironomycin sequestered iron into lysosomes and subsequently reduced mitochondrial iron load, promoting the recruitment and non-canonical activation of BAX/BAK in AML *in vitro* and *in vivo* models. Crispr Cas9 screens uncovered the key metabolic and mitochondrial factors regulating this modality of non-canonical RCD^[53]^.

#### Agents targeting extrinsic pathway

TRAIL Agonism

Agonists of the TNF-related apoptosis-inducing ligand (TRAIL) receptors (DR4/5) have been tested in AML, but response rates are low^[13]^. Novel antibodies against TRAILR1 and TRAILR2 have shown promising preclinical data along with synergy with VEN and are currently tested in phase 1 [Table 2]^[89]^. Eftozanermin alfa (ABBV-621), a second-generation TRAIL receptor agonist binding to the death-inducing DR4 and DR5 receptors, is currently being tested in solid tumors and hematological malignancies [Table 2]^[90,91]^.

FLIP inhibition

FLIP (Fas-associated death domain (FADD)-like IL-1β-converting enzyme-inhibitory protein) is a multifunctional protein that plays a role in regulating the death-inducing signaling complex (DISC) and caspase 8 activation. CDK9 inhibitors and bromodomain inhibitors such as JQ1 have been shown to effectively decrease FLIP expression, leading to enhanced sensitivity to TRAIL-induced cell death in cancer^[92]^. Second-generation FLIP inhibitors have shown preclinical activity in multiple cancer cell lines including AML, and have a high potential for synergy with other apoptosis-targeting agents [Table 2]^[93,94]^.

XIAP inhibition

IAPs act as antiapoptotic proteins by inhibiting caspases and are promising therapeutic targets in AML. Inhibition of XIAP has been shown to sensitize AML cells to chemotherapy or BCL2 inhibitors^[95,96]^. Inhibition of XIAP by antisense strategies or peptides that bind and inhibit the BIR3 domain of XIAP has been tested in phase 1 studies^[97-99]^ but failed in phase 2^[100]^. The SMAC/DIABLO mimetics (SM) birinapant is one of the most clinically advanced SM and is currently being tested in clinical trials for the treatment of certain solid and hematological cancers^[101]^. Birinapant showed limited efficacy as a single agent^[102]^. In a phase 2 randomized, double-blind study, birinapant plus AZA was not superior to AZA alone in advanced myelodysplastic syndromes (MDS) or chronic myelomonocytic leukemias^[103]^. The drug is also being tested in combination with chemotherapeutic agents and immune checkpoint inhibitors. Preclinical data also indicate that combination of Birinapant plus the multidrug resistance receptor 1 (MDR1) may circumvent birinapant resistance in AML^[104]^. ASTX660/Tolinapant is a dual antagonist of XIAP and cIAP is currently under investigation in phase 1/2 studies in solid tumors and in combination with hypomethylating agents in AML^[105,106]^. LCL161, an oral SMAC mimetic, has been tested in patients with myelofibrosis and showed a 30% objective response rate in a recently published phase 2 trial [Table 2]^[107]^.

### Necroptosis inducers

SMAC mimetics combined with caspase 8 inhibition have been shown to trigger necroptosis in AML preclinical models^[108-110]^. RIPK1 inhibition enhanced the therapeutic efficacy of the HDAC inhibitor chidamide in FLT3-ITD positive AML, both *in vitro *and *in vivo*^[111,112]^ and increased the efficacy of differentiating agents^[113]^. Other SMAC mimetics in combination with cytarabine or HMA also showed interesting preclinical results^[114]^. Therapeutic opportunities for cancer cell death induction through endogenous retroelements (EREs) reactivation have been recently described^[115]^. EREs are transcriptionally silent within mammalian genomes due to epigenetic mechanisms. Anticancer therapies targeting the epigenetic machinery reinduces ERE expression, inducing antiviral responses associated with consistent increased phosphorylation of RIPK1/3 and MLKL kinases associated with features of necroptosis in treated tumor cells [Table 2]^[116]^.

### Ferroptosis inducers

Ferroptosis inducers (FINs) belong to four classes^[117]^. Class I FINs include pharmacological agents that limit intracellular glutathione (GSH) through the inhibition of the system x_c_^- ^cystine/glutamate antiporter, as has been shown in non-leukemic models^[28]^. The main molecules of this class, which are active in AML models, are erastin and sulfasalazine^[118,119]^. Class II FINs directly inhibit the detoxifying enzyme GPX4. The lead compound in this class is RSL3 which covalently binds to GPX4. The antitumoral effect of GPX4 disruption has been shown in various models, including AML^[120,121]^. The FDA-approved alkylating agent altretamine has been shown to directly inhibit GPX4^[122]^. Class III FINs target GSH synthesis or cysteine synthetase such as buthionine sulfoximine (BSO), an irreversible inhibitor of rate-limiting enzyme in GSH synthesis, and also cisplatin^[123]^. Focusing on AML, APR-246, a drug known to restore the wild-type conformation of mutant TP53 protein, was shown to actually target GSH metabolism^[124,125]^. Class IV FINs disrupt the balance of iron metabolism and cellular reactive oxygen species (ROS). Dihydroartemisinin (DHA) belongs to this class^[126-128]^, as well as drugs interfering with the antioxidant system CoQ10^[37,38]^ or drugs modulating PUFA metabolism as they are highly sensitive to lipid oxidation [Table 2]^[129,130]^.

Iron chelation and overload play a crucial role in MDS and AML. Therapeutic interventions that modulate iron content and balance within blast cells are at least partially inducing ferroptosis^[131]^. Our group described in detail the mechanism of action of ironomycin that specifically sequesters ferrous iron into the lysosomes, and induces lipid peroxidation and cell death in several models of cancer stem cells as well as in AML through mitochondrial metabolism disruption^[53,54,132]^.

### Agent regulating autophagy

Autophagy deregulation in AML is well documented, but its impact on leukemogenesis remains unclear^[133,134]^. For example, in AML harboring an FLT3 internal tandem mutation (ITD), mTORC1 activation downstream the RET receptor tyrosine kinase suppresses FLT3 protein autophagic degradation^[135]^. In contrast, blocking autophagy in FLT3-ITD AML can increase the survival of mice with FLT3-ITD-driven AML^[136]^. These ambiguous results indicate the complexity of therapeutic intervention based on autophagy in AML. Another layer of complexity lies in the fact that autophagy is a dynamic process. For instance, blocking autophagy cargo can be done at earlier phases (autophagosome biogenesis) or at the later steps (endosome-lysosome fusion).

#### Autophagy inhibitors

Autophagy inhibition can be achieved by blocking the LC3 interacting regions (LIR) that orchestrate diverse stages of autophagy. This strategy was shown to sensitize cytotoxicity to cytarabine^[137]^. Late-stage autophagy inhibitors hydroxychloroquine and/or bafilomycin A1 (BafA1) block lysosomal acidification and are active on leukemia blasts and also sensitize cells to chemotherapy^[138]^. A randomized phase 2 study was recently published testing the addition of hydroxychloroquine to Imatinib in chronic myeloid leukemia and found no significant differences between the two arms^[139]^. SAR405, a highly potent small-molecule inhibitor of the phosphatidylinositol 3-kinase catalytic subunit type 3 (PIK3C3)/Vps34, induces a blockage at the late endosome-lysosome step autophagy flux and shows interesting preclinical efficacy in FLT3-ITD AML [Table 1]^[140,141]^.

#### Autophagy inducers

The mTORC1/S6K1 pathway is critical for the regulation of autophagy in AML initiation and progression, as reviewed by Ghosh & Kapur^[142]^. Therefore, the most studied drugs are the mTORC1 inhibitor sirolimus and everolimus. Despite promising data in preclinical models, clinical studies are ambiguous. Sirolimus combined with the chemotherapy regimen showed a high response rate in patients with baseline mTOR activation^[143]^. In a phase 1b study from the GOELAMS, everolimus plus chemotherapy improved the clinical outcomes of patients with AML^[144]^. But mTOR inhibition plus conventional chemotherapy did not show a clinical benefit in two other studies^[145,146]^. Therefore, mTOR inhibitors still need to be evaluated in AML, and several clinical trials are ongoing (NCT00819546, NCT02638428, NCT01869114).

#### Pharmacological induction of other cell death pathways

Small-molecule inhibitors of the serine dipeptidases DPP8 and DPP9 (DPP8/9) have been shown to induce pyroptosis in human myeloid cells, including cell lines, primary AML samples and mice models of human leukemia^[147]^. Constitutive innate immune activation is a pathogenetic driver of ineffective hematopoiesis and MDS, in particular through the NLRP3 inflammasome^[148,149] ^Recent studies found that the inflammasome can be induced by gene mutations involving mRNA splicing by induction of cyclic GMP-AMP synthase/stimulator of IFN genes (cGAS/STING)^[150]^. As a consequence, the use of immunotherapies targeting inflammatory responses is the subject of many preclinical and clinical studies^[151]^. Mitotic catastrophe is induced by pharmacological agents targeting the mitotic machinery. Cell cycle inhibitors are currently in development in various hematological diseases, including AML^[152,153]^.

## UPCOMING CHALLENGES FOR TARGETING RCD IN AML

The key objectives of translating preclinical results into the clinic are to identify drugs that will treat resistant cells by inducing RCD, detect the population of patients who will respond to a given RCD and design new RCD inducers with high efficacy and low toxicity.

### Target resistances with RCD

Cells resistant to treatment, called “persister cancer cells”, are largely responsible for relapse^[154]^. These cells develop antiapoptotic mechanisms as well as altered metabolism rendering them more susceptible to alternative RCD, in particular autophagy or ferroptosis that are directly related to the metabolic state of the cells^[155,156]^. For instance, preclinical data showed the x_c_^-^ inhibitory activity of salazopyrine and its efficacy against primary AML samples in *ex vivo* cultures and in patient-derived AML models^[118]^. These preliminary results led to the hypothesis that the drug could be repositioned in AML. A clinical trial testing the addition of salazopyrine to ICT in older AML patients, named SALMA (EUDRACT no: 2022-001269-11), will be enrolling soon. Another example is the resistance to VEN, which is mediated through various mechanisms, including BCL2 family protein expression and occupation (MCL1 dependency), cellular differentiation state (monocytic versus stem cell-like), cellular metabolic state or sensitivity to mitochondrial machinery disruption^[11]^. One of the major limitations that emerged from both *in vitro* and clinical studies with the BH3-mimetics is the low sensitivity of TP53 mutated blasts to this class^[157-159]^. Strategies of treatment that overcome TP53-dependent apoptosis can be used, such as ironomycin that directly activates BAX/BAK in a BCL2-family protein-independent manner^[53]^. Optimized clinical-grade derivatives of ironomycin with better therapeutic windows are currently under development^[160]^.

### Identify new biomarkers

The second challenge is to identify the population of patients who will benefit from RCD inducers. Recent findings showed that ferroptosis-associated gene signatures can be assessed by transcriptomic methods such as RNA sequencing. These signatures predict survival and could possibly guide therapeutics by selecting patients who could be treated by the use of ferroptosis inducers^[161-164]^. Necroptosis transcriptomic signatures have also been found in MDS, indicating that such predictors could identify patients who would benefit from necroptosis inducers^[165]^. Functional assays such as BH3-profiling have been shown to be a highly efficient companion test that can predict response to the BH3-mimetics inhibiting BCL2 family proteins^[20,166]^. Our team published a translational proof-of-concept study in which relapsed or refractory AML patients were selected according to molecular and *ex vivo* drug sensitivity profiles^[167]^. Future clinical trials using companion tests based on molecular or functional approaches will confirm the feasibility and efficacy of this strategy.

### Discovery of new RCD inducers

The use of targeted therapies has transformed AML treatment. Small molecule inhibitors of genes that underwent common somatic mutations, such as FLT3 or IDH inhibitors, have been approved recently for AML relapsed or refractory patients^[168]^. However, most of the patients cannot benefit from targeted therapies, because of a lack of targetable genetic alterations. On the contrary, BCL2 inhibition success story is based on the AML blast dependency to apoptosis independently of AML targetable mutations. Recent findings showed that AML cells are sensitive to ferroptosis induction, suggesting that efforts should be made to develop new ferroptosis inducers^[169]^. A validated approach is to use an unbiased strategy by performing high throughput screening of chemical libraries for their ability to induce ferroptosis (or other RCD) *in vitro* and *in vivo*^[122]^. In parallel, a better understanding of the ferroptotic molecular pathways can be obtained using CRISPR Cas-9 resistance screens of a given ferroptosis inducer that will identify crucial genes involved in cell death. We recently used this strategy to uncover the molecular mechanism of ironomycin^[53]^. This integrative strategy will help to design new tailored drugs and pave the way for future clinical trials based on RCD.

## CONCLUSION

Treatment of AML has remained unchanged for more than 40 years with a one-size-fits-all intensive chemotherapy approach for eligible patients. In 2017, the FDA approved the utilization of two targeted therapies for patients with a molecular alteration in FLT3 or IDH genes^[168]^. Another significant advancement was made in 2020 with the publication of the VIALE-A study reporting the efficacy of VEN for the treatment of AML patients who are not eligible for intensive treatment^[10]^. Despite higher response and survival rates than before, there is still improvement to be made in the management of AML patients. A better understanding of molecular RCD pathways in collaboration with integrative translational studies will allow the design of new drugs or the repositioning of older ones to overcome resistance in AML.

Intrinsic apoptosis is initiated by the formation of pores permeabilizing the outer membrane of mitochondria (MOMP) induced by the oligomerization of BAX recruited from the cytosol and BAK inserted at the mitochondrial membrane. BAX and BAK interact with a series of antiapoptotic inhibitors proteins (BCL2, BCLXL, MCL1) and proapoptotic activator proteins (BIM, PUMA, NOXA, BID). MOMP induces a release of cytochrome c that cleaves caspase 9 and subsequently activates the effector caspases 3 and 7, leading to cell death. Extrinsic apoptosis is triggered by the ligation of cell death receptors (FAS, TRAIL and TNFR), which allow the assembly of the “death-inducing signaling complex” (DISC) and the subsequent activation of caspase 8. Caspase 8 induces the cleavage of BID, leading to the activation of BAX and intrinsic apoptosis and the direct activation of caspases 3 and 7, finally leading to cell death. Inhibitors of apoptosis (IAP) family, including XIAP, cIAP1 and cIAP2, are inhibitors of caspase 9 and the death receptor TNFR, whereas FLIP is the inhibitor of caspase 8. SMAC/DIABLO is a major inhibitor of IAPs. Necroptosis is induced by the ligation of cell death receptors and the activation of the receptor-interacting protein RIPK1 and RIPK3 that interacts with protein complex mixed lineage kinase domain-like pseudokinase (MLKL). Phosphorylation of MLKL results in the formation of pores on the cell membrane, followed by cell death. IAPs and Caspase 8 inhibit RIPK1 activation and block necroptosis. Ferroptosis is the result of lipid peroxidation triggered by the direct inhibition of the antioxidant enzyme GPX4 or by the blockage of the x_c_^- ^cystine/glutamate antiporter (xCT-). Depletion of cysteine import and intracellular glutathione (GSH) increases lipid peroxides which is exacerbated by the Fenton reaction, where H_2_O_2 _and iron react to generate hydroxyl radicals. Autophagy is a process in which cytoplasmic components, including intracellular organelles, are degraded by the lysosomes. The first step of autophagy is the formation of phagophores, followed by the generation of double-membrane autophagosomes regulated by autophagy-regulated genes (ATG) and lysosomal fusion. Autophagy blockage or excess can induce cell death.

## DECLARATIONS

### Copyright

© The Author(s) 2023.
